# Diabetes mellitus associated neurovascular lesions in the retina and brain: A review

**DOI:** 10.3389/fopht.2022.1012804

**Published:** 2022-10-31

**Authors:** Stephen H. Sinclair, Elan Miller, Kiran S. Talekar, Stanley S. Schwartz

**Affiliations:** ^1^ Pennsylvania College of Optometry, Salus University, Philadelphia, PA, United States; ^2^ Division of Vascular Neurology, Vickie & Jack Farber Institute for Institute for Neuroscience, Sidney Kimmel Medical College (SKMC) Thomas Jefferson University, Philadelphia, PA, United States; ^3^ Department of Radiology, Section of Neuroradiology and ENT Radiology, Clinical Functional Magnetic Resonance Imaging and Diffusion Tensor Imaging at Thomas Jefferson University Hospital and The Jefferson Integrated Magnetic Resonance Imaging Center (JIMRIC) Sidney Kimmel Medical College at Thomas Jefferson University, Philadelphia, PA, United States; ^4^ Department of Endocrinology and Medicine, University of Pennsylvania, Philadelphia, PA, United States; ^5^ Main Line Health System, Philadelphia, PA, United States

**Keywords:** systematic autoimmune disease, new treatments and technology, neurovascular alterations, new diagnostic and therapeutic options, diabetic retinopathy

## Abstract

Diabetes mellitus (DM) is now recognized as a system-wide, autoimmune, inflammatory, microvascular disorder, which, in the retina and brain results in severe multifocal injury now recognized as a leading cause, world-wide, of progressive vision loss and dementia. To address this problem, resulting primarily from variations in glycemia in the prediabetic and overt diabetic states, it must be realized that, although some of the injury processes associated with diabetes may be system wide, there are varying responses, effector, and repair mechanisms that differ from organ to organ or within varying cell structures. Specifically, within the retina, and similarly within the brain cortex, lesions occur of the “neurovascular unit”, comprised of focal microvascular occlusions, inflammatory endothelial and pericyte injury, with small vessel leakage resulting in injury to astrocytes, Müller cells, and microglia, all of which occur with progressive neuronal apoptosis. Such lesions are now recognized to occur before the first microaneurysms are visible to imaging by fundus cameras or before they result in detectable symptoms or signs recognizable to the patient or clinician. Treatments, therefore, which currently are not initiated within the retina until edema develops or there is progression of vascular lesions that define the current staging of retinopathy, and in the brain only after severe signs of cognitive failure. Treatments, therefore are applied relatively late with some reduction in progressive cellular injury but with resultant minimal vision or cognitive improvement. This review article will summarize the multiple inflammatory and remediation processes currently understood to occur in patients with diabetes as well as pre-diabetes and summarize as well the current limitations of methods for assessing the structural and functional alterations within the retina and brain. The goal is to attempt to define future screening, monitoring, and treatment directions that hopefully will prevent progressive injury as well as enable improved repair and attendant function.

## Introduction

There are presently 463 million persons worldwide with diabetes mellitus, representing 9.3% of the adult population, with an expected doubling in the next 25 years (1), and with approximately 37% of US adults older than 20 years and 51% older than 65 having prediabetes with predicted high rates of conversion to type 2DM with passing years (2). Because of this, prediabetes, along with diabetes, are now recognized as a major risk factor for the development and progression of retinal and brain neurovascular injury (3–7), resulting in severe vision loss (8) as well as moderate vascular cognitive impairment with progression to dementia (4, 7, 9). The focus of the medical industry has predominantly been to examine the effect of control of hyperglycemia along with other aggravating factors (hypertension, smoking, obesity, and hyperlipidemia) on the progression of large vessel occlusive disease, because of the rising incidence of stroke and myocardial infarction (10). However, it is now recognized that the small vessel disease is 20-30 times more common resulting in severe disabling neurovascular unit injury with resultant progressive vision and cognitive impairment (11–19).

To address this problem it must be realized that, although some of the injury processes associated with diabetes are system-wide and, therefore, possibly amenable to systemic therapy, there are varying response, effector, and repair mechanisms that differ from organ to organ or within varying cell structures within an organ tissue. The inflammatory processes in the pancreas that kill the beta cells, resulting in reduced insulin production, are different from those in the adipose tissue that increase insulin resistance, and are also different from responses within tissues of neurons, astrocytes, microglia, pericytes and endothelia of the retina and brain, now termed the “neurovascular unit”. This review article will summarize those processes currently understood within the retina and brain and review current systemic methods to potentially manage those problems, preventing progressive injury as well as enabling or improving repair. This article will also summarize current methods for assessing those clinical structural and functional alterations within the retina and brain that would enable, not only improved outcomes in the clinic through earlier, more effective, interventions, but also the recognition of the limitations of the evaluation methods and their application.

## The normal human neurovascular unit within the retina and brain and the imaged lesions associated with diabetes

The inner retina, as well as the subcortical, deep, white matter of the brain share a common organization of the microvasculature, supplied by small perforating independent vascular units of similar components, that demonstrate little ability to provide collateral flow (20, 21). They also demonstrate a similar intensive balance between tissue metabolism and vascular regulation of blood flow, termed autoregulation. In the inner and middle retinal neuron layers the local precapillary arterioles are very sensitive to local extravascular oxygen levels. Because of the tendency of the intense light to potentially generate oxygen free radicals within the tissue (22), the blood flow is maintained at critically low oxygen levels. Therefore, the retina requires careful regulation of flow, moment to moment, despite changes in arterial inflow or venous outflow pressures. While the choriocapillaris supply to the outer retina manifests a high flow rate as it provides high oxygen delivery for the intense oxygen consumption of the immediately overlying photoreceptors as well as a stabilizing heat sync, little oxygen diffuses through into the middle and inner retina. At night the photoreceptor and middle nuclear layer cells are hyper-polarized (termed “dark current”) to reduce spontaneous transmissions (flashes of light), with adjustments matched by inner retinal flow. In humans, non-invasive flow measurements allow assessment of baseline flow by laser doppler velocimetry along with autoregulatory changes induced by increased or decreased inhaled oxygen and perfusion pressure (23, 24).

Astrocytes within the inner retina synapse on blood vessels to provide the autoregulation (25), while Müller cells, which span all retinal layers, coordinate the vascular responses to meet the metabolic demand of neurons, interchange metabolites, recycle neurotransmitters and glutamate, and control extracellular ion homeostasis. Microglia, which normally reside within the plexiform retinal layer, exhibit ramified processes responsible for immune surveillance along with monitoring noxious insults such as oxidative stress, hypoxia, or inherited mutations, all of which trigger proliferation and migration to the sites of injury (26, 27). Coordinated activity among neurons, Muller cells, astrocytes, and microglia, together with the microvasculature blood supply, therefore, is essential for the maintenance of normal metabolic function and vision. Although the local autoregulatory mechanisms pre-eminently attempt to maintain flow aligned with metabolism, it is recognized that certain conditions, such as peripheral limb, cold-induced vasospasm also produce coexistent transient reductions in retinal blood flow from focal arteriolar vasospasm in susceptible individuals (termed systemic vascular dysregulation, SVD) (28). Although these are thought to be transient, SVD has been associated with an increased risk of anterior ischemic optic neuropathy, retinal venous occlusion, central serous chorio-retinopathy, and especially for glaucomatous disc nerve-fiber-layer loss (29, 30). More recently SVD has been associated with a reduced parapapillary microvascular density in what were thought to be normal eyes (30), indicating that the ocular abnormalities may represent only a visible portion of a system-wide disorder that, alone, or perhaps in conjunction with other abnormalities, may result in more prolonged and perhaps progressive injury to the microvasculature of the eye, brain, and other organs.

The retina and brain manifest the coordination of blood flow to metabolic demand, synaptic activity, and waste removal, coordinated through neurotransmitter-mediated signaling, particularly through glutamate release of nitric oxide from neurons and of arachidonic acid derivatives from astrocytes (and possibly from neurons) and by adenosine tri-phosphate (ATP) conversion to adenosine (31–34). However, the relative importance of the neuronal and astrocyte pathways have been demonstrated to differ across brain areas (35). Consequently, the relationship of blood flow to the underlying neural activity will differ, implying that functional imaging signals arising from these pathways will also reflect different aspects of neuronal function. It was initially thought that most of the energy utilized with neuronal activity was derived from glycolysis rather than oxidative phosphorylation, because neuronal activity was observed to increase glucose uptake much greater than O2 uptake. However, later studies have observed less of a difference, indicating that nearly all of the ATP is generated by oxidative metabolism of glucose (36) to glutamate with flow increases paralleling synaptic activity (37).

Within the retina the blood-retinal barrier is maintained by a continuous microvascular endothelium and its underlying basement membrane with pericytes that tightly encircle the endothelium, and astrocytes within the surrounding tissue space that extend their cell processes towards the endothelium to insert on the basement membrane. Pericytes are noted to be highly susceptible to damage in ischemic conditions (when ATP levels are low) suggesting the possibility that pericytes are the cause of constricted capillaries observed at the start of a stroke (38). The astrocytes remain in rigor (because no ATP is available to relax their contractile filaments), causing the capillaries to remain too narrow for the passage of blood cells, predominantly leukocytes. In agreement with this, pericytes are noted to remain constricted even for hours after the re-opening of an occluded parent artery (in brain models (39)) resulting in endothelial damage and capillary leakage. Suppression of the oxidative and nitrosative stress prevents this pericyte constriction, restoring the patency of capillaries and tissue recovery (39). This has important implications to the understanding of microvascular responses to prolonged ischemia due as well to small vessel disease, as well as with the occurrence of vacillations in oxygen levels or in blood pressure (as occur with sleep apnea in which a sudden drop in oxygen induces autoregulatory dilation of the retinal arterioles resulting in capillary hypertension that is severely aggravated with the sudden rise in blood pressure occurring at the “reprise” end of each apneic episode and has been associated with capillary occlusion even in normal, non-diabetic individuals (40).

In the diabetic individual the mechanisms by which the retinal microvasculature is more susceptible to large, as well as small vessel abnormalities of flow regulation are now better understood. Increased blood flow has been measured within the retina associated with elevations of serum glucose occurring in the “prediabetic” as well as diabetic with variable hyperglycemia (41). This results in capillary hypertension (42, 43) along with a reduced capability of autoregulation (23, 43–45), apparently worse in the middle retinal layers than that in the innermost layers (46), but, in both, due to abnormalities of nitric oxide (NO) within the arteriolar musculature (6).These mechanisms, as well the recognized additional factors of hypertension, smoking, sleep apnea, and others that will be discussed below, result in aggravation of both retinal small vessel ischemic lesions in the diabetic and prediabetic populations (9, 47) with progressive vision loss [and in the brain which shares similar mechanisms and outcomes resulting in microvascular cognitive impairment and dementia (48)]. While arteriolar oxygen reactivity and its match with metabolism have been the primary focus of investigations into the aberrant cause of the retinopathy, it is also now recognized that toxic byproducts accumulate in the interstitial, perivascular space resulting in neuronal apoptosis. Such toxic products were thought to be eliminated primarily *via* venular outflow. However, more recently perivascular fluid outflow has been proposed to exit also *via* the lamina cribrosa and a hypothesized glymphatic cerebrospinal fluid (CSF) drainage (49). While the importance of this aspect of toxic removal is now better recognized in the pathogenesis of glaucomatous nerve fiber layer injury, its causation in other retinal and brain neurodegenerations at this time remains only hypothetical.

Diabetic retinopathy (DR) has traditionally been considered to be a microcirculatory disease caused by the deleterious metabolic effects of hyperglycemia *per se* and the metabolic pathways triggered by hyperglycemia, including the polyol (50), hexosamine, and diacylglycerol-protein kinase C (DAG-PKC) pathways (51, 52), that result in advanced glycation end-products (53) and the induction of oxidative stress (54). The primary lesions in both the retina and brain, however, are now recognized as small, neurovascular lesions composed of both focal vascular occlusions mixed with varying degrees and types of inflammatory endothelial and pericyte injury. These produce small vessel leakages that result in injury to structural astrocytes, Müller cells, and microglia, with both processes causing progressive neuronal apoptosis that occurs in both prediabetic and diabetic individuals. The earliest structural change appears in both organs to be a loss of microvascular pericytes *via* apoptosis or migration, which then leads to weakening of the blood-retinal barrier (55) through loss of the inter-endothelial tight junctions (56). This precedes, but results, as well, in apoptosis of the endothelial cells, resulting in loss of endothelial nitric oxide (NO) production, the primary vasodilator that provides for the normal small arteriolar autoregulatory capabilities discussed above. Furthermore, capillary hypertension, caused by the elevated flow levels in the remaining patent vessels, is recognized to stimulate endothelial cell production of inter-cellular adhesion molecules (ICAMS) that slow and obstruct the normal passage of leukocytes (also known to be stiffened, less deformable, in the diabetic) (57) with subsequent breakdown and endothelial injury (58, 59). Studies of arterial and venular oxygen saturations indicate reduce oxygen delivery within the retina (60), confirming the secondary effects of such focal occlusions that occur with the secondary increased flows within the microvasculature remaining patent, but with poor localized oxygen delivery.

For the past 50 years, however, the clinical focus for the detection and grading of diabetic retinopathy, as well as for considering treatment, has been the viewable secondary retinal vascular lesions of microaneurysms, hemorrhages, intra-retinal and epiretinal microvascular proliferation, arterial wall thickening and venular irregular dilation, with varying degrees of intracellular and extracellular edema and lipid (61, 62). It is now recognized, however, that neuronal and Muller cell death occurs in focal patterns within the retinal ganglion cell and middle nuclear layers, as well as photoreceptors much earlier (in both diabetic and prediabetic individuals), even prior to the pericyte and endothelial cell apoptosis (63–71). These alterations, however, are not visible on examination or appreciated with standard imaging by fundus cameras or ocular coherence tomography (OCT) devices until there is fairly severe, widespread, neuronal death and atrophy that is appreciated structurally on OCT as progressive thinning of the inner retinal nuclear layers and nerve fiber layer (67–70, 72–74) and occurs in 20% of diabetics even prior to observation of microaneurysms (72) and in prediabetics as well (71). The Müller cells (macroglia) and microglia also appear to play a key role in what is considered to be an inflammatory process through the expression of vimentin and glial fibrillary acidic protein (GFAP, an intermediate filament-III protein uniquely found in astrocytes) that results in inhibition of both neural and capillary regeneration (75–77). In addition, it is now recognized that Müller cells act as living optical fibers which guide red and green light through the inner retinal tissue to specialized cone photoreceptors (78), minimizing intraretinal light scatter to maintain the critical spatial distribution of light patterns (79) all of which support high acuity visual perception (80). Therefore, reactive gliosis of Müller cells (75) would contribute to the early visual abnormalities that have been detected in diabetic subjects *via* multifocal ERG (81, 82), or when tested at fixation with blue-sensitive acuity or contrast sensitivity at low light levels (66, 83–85) or with resolution perimetry conducted under low illumination and low contrast conditions (86).

Unfortunately, these imaging methods and functional tests are seldom performed in the standard ophthalmologist’s or optometrist’s office and are not recommended in current evaluation guidelines for diabetics or prediabetics (87), resulting in the relatively late discovery (88) when the more severe lesions are observed. This delays intervention, currently consisting primarily of repeated intravitreal injections of antiVEGF antibodies that result in marginal vision improvement. In multiple studies, only 25– 34% demonstrate improvement of ≥3 lines of the ETDRS chart acuity, and primarily occurring only in the eyes with severe retinopathy and poor initial vision, and with 23% considered non-responders and 27% having only a moderate response (62, 89–92). Recent methods of OCT derived microvascular analysis (termed OCTangiography, OCTangio) have provided imaging analysis of the inner retinal microvasculature. These demonstrate that reductions in microvascular density occur within the macular retina and radial peripapillary capillaries prior to the development of the traditional retinopathy lesions and progressively worsen over time, correlated with worsening grades of the retinopathy (93–98) and with inner retinal apoptotic neuronal atrophy (99–102). However, whether the microvascular occlusions, with degradation of the endothelia, occur prior to, and cause, the neuronal apoptosis, or whether the vascular changes are the consequence of prior neural tissue inflammation and cellular injury are still to be answered. What are required are improved methods to diagnose and quantitate the focal microvascular injury with overlaid neuronal apoptosis imaging and functional testing that expand beyond the current limitations to empower the clinical evaluation of evolving treatments discussed below (86) (103–107).

Similar problems have limited the imaging and functional evaluation of the effects of diabetes within the brain. The primary focus over the last 30 years has been on defining the risk for development of large vessel obstruction because of the acute nature of a stroke occurrence and the disability that results (4, 6). However “silent strokes” of the cerebral white and deep gray matter served by the perforating cerebral arterioles occur more commonly. Such ischemic lesions are most often demonstrated by subcortical white matter hyperintensities (WMHI’s)-defined as a focal T2-hyperintensity and T1-hypointensity lesions larger than 3 mm on Fluid Attenuated Inversion Recovery (FLAIR) MRI (108), and are often observed with microbleeds or lacunes (12, 109). These WMHI’s are designated “neuroradiological markers of cerebral small vessel disease” and have become the focus of intense study (108–112). In addition, there are numerous reports, reviews, and meta-analyses that have been published evaluating the relationships between the cerebral radiological markers of small vascular lesions and the associated retinopathy lesions. In patients with notable retinopathy, post mortem vascular lesions in the brain have been observed to correlate with the described WMHI’s (6, 16) as well as with lacunar infarcts (6, 113, 114), non-lacunar infarcts (14), cerebral microbleeds (115), symptomatic intracranial large artery disease (21), and the severity of cognitive disorders (6, 116–119). They are also associated with negative health outcomes, including stroke related disability and mortality (109, 120).

Although the major goals in this field encompass developing a precise understanding of their pathogenesis and identifying potential prevention and treatment targets, many barriers to these goals remain unaddressed (109, 112). The neuropathological substrates differ among the various localizations of these WMHI lesions (121, 122) with the subcortical WMHIs appearing as isolated foci in the superficial white matter and which, on occasion, may appear with small hemorrhages. In most cases, these superficial cortical lesions are not contiguous with those located periventricular, indicating, therefore, potentially different risk factors for and effects upon cognition (123). Periventricular WMHI lesions, at the rims or caps, tend to be non-progressive and have the greatest consensus regarding their neuropathological correlates. They consist of a denuding of the ependyma from the ventricular lining with variable sub-ependymal gliosis (124). Furthermore, among the neuroradiological evaluations of such lesions, some may remain stable over time while others progress or, occasionally, may regress. Studies indicate that infarcts (particularly the lacunar variety) are present in a sizable proportion of those with progressive cognitive impairment, with the lesions often antedating the cognitive decline. Although lacunar infarcts and microinfarcts are unlikely themselves to be directly responsible for the decline in most patients, they may serve as markers of overall cerebral neurovascular unit hypoperfusion that can result in the progressive decline to more diffuse brain damage that, as yet, is undetectable by radiologic methods (124). To some degree, however, this is refuted by the study of Launer et al. (125), and in addition, novel radiologic markers have recently been developed that appear to be more direct measures of vascular integrity. For example, endothelial and pericyte dysfunction, as noted above, causing small vessel flow abnormalities within the retina, can be evaluated in the brain by the measurement of cerebral blood flow regional inhomogeneity (126) at rest and in response to specific challenges (such as breath holding or hypercapnia) (110) or by the recognition of micro hemorrhages occurring within the identified lesions (127). MRI data suggest, as well, that leakage of an injected, intra-vascular, contrast marker of blood-brain barrier dysfunction, is associated with the WMHI’s and small vessel derived cognitive impairment (123, 128–130). There is ample evidence that such lesions affect neuronal networks involved in cognition, memory, and behavior (thalamo-cortical, striato-subfrontal, cortico-subcortical, and limbic systems (15)). However, progress in understanding the lesions has been slow in dementia research because investigators have concentrated predominantly on whole brain atrophy, which, although correlating with global cognitive performance, precludes evaluation of the selective effects of the position or volume of the hyperintensities upon specific cognitive task performance. The spatial distribution of cortical small vessel WMHI’s does explain some of the clinical manifestations. The WMHI’s and lacunes in the frontal, subcortical areas are of concern because of the role of these areas in executive function (21, 131) and decision making (132). Likewise, WMHIs in the periventricular areas, where long connecting cortico-to-subcortical tracts gather, can explain reductions in processing speed. Executive dysfunction is a hallmark cognitive feature, comprising deficits in information-processing speed, psychomotor efficiency, attention, cognitive flexibility, and visuospatial perception. Dysfunction in these domains can impair planning and problem solving (133, 134) that are of significant concern, especially in the elderly because they can affect daily living independence. Moreover, executive dysfunction is a well-established precursor to dementia, depression, disability, and mortality.

Therefore, although WMHI volumes explain only a small degree of cross-sectional variation in cognition and cognitive decline (135) longitudinal studies in diverse populations do consistently demonstrate that increasing WMHI’s overall summed volume does predict general cognitive decline and incident dementia (136), as well as a greater than a 3X increased risk of the occurrence of a subsequent stroke (108, 123, 130, 137).

## Pathologic mechanisms implicated in the development of brain and retinal lesions in diabetic patients

Review of the radiological and histopathological characteristics of cerebral small vessel disease, as noted above, suggests that the understanding is primarily limited to abnormalities of brain parenchymal injury, specifically that of white matter, rather than the actual small vessel causation or the associated pathology. It has been generally assumed that such parenchymal abnormalities are secondary to the small vessel disease, although, as in the retina, both the microvascular or parenchymal initiation pathways are plausible. Certainly the components of the vessels and those of the surrounding tissue are so tightly interconnected that affecting one will very likely rapidly affect the other. Under physiologically normal conditions, cerebral blood flow autoregulation, similar to that within the retina, relies on a number of molecular pathways that protect the microcirculation from extreme variations in pressure as well as oxygen and metabolite concentrations. Chronologic aging of the vessel wall components and/or exposure to risk factors (hypertension, hyperglycemia, dyslipidemia, sleep apnea) can compromise the structure and functionality of such controlling vessels, and with ongoing oxidative stress, may cause further tissue and microvascular injury, which, in turn, disrupts their autoregulation capacity, aggravating the injury in a downward spiral. Furthermore, hyperglycemia, as well as glycemic vacillation, and insulin resistance are known to trigger inflammatory and oxidative pathways, including dysregulation of nitric oxide production with less bioactivity and the production of reactive oxygen species (ROS), toxic metabolites, and advanced glycation end products (138, 139). While all are involved in the microvascular injury and tissue destruction, this review will limit the discussion. To better understand the various associated mechanisms, the reader is referred to additional reviews by ourselves and others (86, 109, 140)

### Microvascular injury, dysregulation and occlusion

One proposed mechanism of vascular injury associated with diabetes is through impairment in the regulation of the transient receptor potential cation channel (TRPC). Under physiologically healthy conditions, the precapillary arterial wall responds to high pressure by upregulating TRPC, triggering reactive vasoconstriction. However, continuous stimulation ultimately may result in poor flow autoregulation to the variation of the stimulant (141). Both aging and deficiencies in insulin-like growth factor-1 (IGF-1) in the presence of hypertension have been shown to impair upregulation of TRPC within the retina (142, 143). The loss of these protective autoregulatory processes leaves the microcirculation vulnerable to the damage caused by the variation of pressures within the microvasculature resulting in increased blood-brain barrier leakage and neuroinflammation (109, 144). There is also a gradual accumulation within the microvasculature of multiple molecular fractures of the intima and internal elastic lamina. The accumulation of such patches eventually transforms the elastic lamina from a fully elastic, homogeneous structure to a stiffened, friable wall, resulting in a reduction in vascular compliance. The resulting array of structural degenerative events in the vessel wall, including death of endothelial cells, basal membrane thickening, and atrophy of the smooth muscle cells (145, 146) can all lead to rupture (microbleeds), microinfarcts, and loss of tight junctions with reduced blood brain-barrier integrity. In the retinal capillaries of the diabetic, leukocyte drag and adhesion occur due to leukocyte elevated stiffness and the upregulation of endothelial derived adhesion molecules (ICAMS and VCAMS) that adhere with the leukocyte surface integrins resulting in the adherence of the leukocyte to the endothelium and degradation producing focal microvascular leakage and occlusion (147–149). The resultant increased permeability of the blood-brain barrier can lead to interstitial accumulation of cytokine proteins with further worsening of inflammatory/oxidative stress events that damage the gray and white matter parenchyma. Pericytes and oligodendrocytes are extremely vulnerable to this ischemic and toxic insult (38) resulting, as well, in the impairment of the tissue repair mechanisms (150).

Diabetes mellitus, therefore, is now recognized to involve a complex, systemic, autoimmune, inflammatory disorder that causes focal microvascular occlusions and alterations of the blood-retinal and blood-brain barriers that occur in “pre-diabetic” as well as diabetic individuals (1, 3, 86). In the Diabetes Prevention Program (DPP), 7.9% of subjects with impaired glucose tolerance had retinopathy (151), similar to the 8.1% prevalence of retinopathy observed among individuals with prediabetes in the Gutenberg Health Study (152) with the variability of the glycemia a significant recognized risk factor for DR development and progression (153). HbA1c has been the primary criteria differentiating the diagnosis of prediabetes from diabetes, but it must be stressed that there are many well-characterized “pitfalls” of this including other systemic illness and hematological disorders that disrupt the reliability of HbA1c as an integrated measure of mean plasma glucose. Although DR or its progression is recognized to be related to HbA1c. However, studies across the globe have observed that the risk of many of the associated co-morbidities are the same in diabetics and prediabetics and affect all age groups indicating these are complicated interactions (4). Across the striae of hyperglycemia definitions, the increasing requirement for insulin, brought about by the progressive insulin resistance occurring in peripheral fat together with the pancreas Beta cell loss (both a product of immunologic reactions) is associated in itself, with aggravated risk of vascular induced retinal as well as cognitive dysfunction (3).

### Inflammatory mechanisms associated with the neurovascular injury

As discussed above, in the diabetic or prediabetic patient, there is chronic systemic low-grade inflammation (86) which is reflected by high levels of serum cytokines such as tumor necrosis factor-alpha (TNF-α), C reactive protein (CRP), and the interlukins, IL-6, iL-18, IL-1B and the receptor antagonist, C5a (154) along with granulocyte and monocyte elevations (140, 155). While this inflammatory state is thought to be the mechanism by which metabolic disorders, such as diabetes. are associated with the development of small vessel associated organ failure, there is significant variability of the cytokines that indicates a complex process (156–158) and which, therefore, will require more complicated design trials to define which will predict the development and progression (or regression) of the neuropathic lesions.

The loss within the retina of the ganglion cell layer neurons with hyperglycemia appears preceded by earlier loss of the microvascular pericytes and then endothelia, as discussed above, that results in loss of reactivity, with early vasodilation that is observed clinically along with early abnormal permeability of the endothelial barriers (42, 55). Chronic, variable hyperglycemia has also been suggested to cause an increase in release of glutamate with loss of neuroprotective factors due to oxidative stress with the accumulation of waste products of glycolysis that is thought to trigger the ganglion cell apoptosis (66). However, we must acknowledge that all of the supporting structures of the neurons, including the Müller cells (macroglia), microglia, and infiltrating monocytes have all been defined as critical actors in this neuroinflammatory process. Activated Müller cells mediate both protective and detrimental effects upon the ganglion cell layer neurons of the retina through a variety of receptors that result in the expression of multiple factors that affect neuronal survival including GFAP, vimentin (159, 160) and growth factors, such as brain-derived neurotrophic factor (BDNF) and platelet-derived growth factor-B (PDGF-B) (161). They also manifest a protective role by absorbing glutamate, reducing the cytotoxic effects of the extracellular glutamate levels (162), providing protection for neuronal growth, survival, and synaptic plasticity while reducing the oxidative stress (64, 163). Diabetes not only reduces the Muller uptake of extracellular glutamate (164) but also reduces the activity of the enzyme, glutamine synthetase, hindering the ability to convert the excess glutamate to inactive glutamine (165) or to oxidize the glutamate to a-ketoglutarate (162). Activated Müller cells, therefore serve not only as rapid sensors of neuronal damage to initiate repair and neural regeneration (166, 167), but they may also initiate a deleterious inflammatory process with the release of proinflammatory cytokines, such as activating transcription factor 4 (results in release of ICAM-1 and vascular endothelial growth factor (VEGF) (75, 168) as well as TNFα, interleukin-1 (IL-1), and other cytokines known to exacerbate the apoptosis of adjacent neurons (65, 161, 169). This dual action, therefore, is certainly a double-edged sword and should be taken into account in the design of therapeutic approaches addressed to abrogate Muller glial activation.

Microglia within the retina and brain are the principal immune effector cells, constantly surveying their environment in preparation to react to insult or injury. Normally phagocytic microglia should clear damaged myelin and allow neuronal repair to proceed. However, a chronic, pro-inflammatory state, such as that induced with hyperglycemia, alters the response, preventing remyelination (170). In human diabetic retinas, activated microglia are often observed to be associated with the vasculature, leading to the term “microglial perivasculitis”, and resulting in the release of numerous cytokines, including IL-6, IL-1B, TNFa, and monocyte chemoattractant protein-1(MCP-1) with several studies indicating these cytokines contribute to the microvascular permeability and occlusive complications as well as progressive neuronal apoptosis (64, 171). It should be noted, however, that activated microglia may also demonstrate a neuro-supportive function, similar to Mueller cells producing anti-inflammatory and neurotrophic factors, including IGF-1, BDNF, and GDNF (glial cell derived neurotrophic factor), among others (172–174).

In the brain, activated microglia and their supportive activities observed in periventricular WMHI’s (136), interestingly, have not been noted in the subcortical WMHI’s (121). Similarly, in the retina a variance has been demonstrated in the reactivity to the ischemia wherein the deeper portion of the microvascular complex, serving the middle nuclear neuronal layer, appears to provide a different reaction to the focal ischemia than within the more superficial ganglion cell layer. Ultimately, the detection of microglial activation may have value in early disease diagnosis, as modulation of microglial responses appears to perhaps provide the ability to alter disease progression. While receiving less attention than microglia, nonetheless mast cells within the brain represent an important source of immune signaling. Mast cells occur within the dura and meninges, and have demonstrated the ability to promote blood-brain barrier breakdown, edema, neutrophil infiltration, and hemorrhage in animal models of focal cerebral ischemia (175, 176). Yet another factor, synthesized, stored, and released by mast cells, and implicated in retinal and brain barrier breakdown is the angiogenic, vascular endothelial growth factor (VEGF) (175, 177) the treatment of which is discussed below.

Hyperglycemia and glucolipotoxicity-induced oxidative stress also drive intracellular changes that result in upregulation of proinflammatory mediators (86, 178, 179). These are generally produced by external toxins, nutrients, etc. that transduce within the cells to cause DNA methylation and histone modification leading to altered gene expression (178, 180). The mediators also cause alterations of microRNAs (miRNA), small, single-stranded non-coding RNA molecules that regulate post-transcriptional gene expression; such alterations may occur even as early as within the fetus of a pregnant diabetic and may accumulate throughout life with recurrent stress-induced influences (86, 181, 182). Such hyperglycemia induced dysregulation occurs within multiple tissues resulting in the established complications of diabetes, in particular endothelial dysfunction associated with retinopathy (183). In particular, miRNA-126, which occurs only within microvascular endothelia, seems to be the miRNA most linked to pathways in the development of both type 1 and 2 diabetes and the tissue complications; since miRNA-126 has been shown to play an important role in maintaining endothelial cell homeostasis and vascular integrity (184, 185), it has been suggested that this unique plasma miRNA might become a valuable tool to predict the micro- and macro-vascular complications. In addition, clinical series have demonstrated that elevated serum levels of multiple other miRNA’s, notably -7,-122, and -210, antedate the development of type 2 diabetes and have also been found significantly associated with the microvascular complications (183), suggesting the possibility they may represent potential targets for evaluation of their sensitivity for predicting and monitoring microvascular injury and repair.

Growth differentiation factor-15 (GDF-15), also known as macrophage inhibitory cytokine-1, has been demonstrated enhanced as a response to tissue ischemia (186). Investigations have shown a positive association between GDF-15 serum concentrations and diabetes risk of retinopathy with higher levels associated in proportion with the grade level of retinopathy (187). However, the mechanisms involved in the pathogenesis remain unclear, although possible explanations have suggested GDF-15 is involved in oxidative stress and endothelial dysfunction (187, 188) as well as in the inflammatory and immune processes (187, 189).

## Effects of traditional hyperglycemia medications on retinopathy and CNS cognitive function

While the Diabetes Control and Complications Trial and the United Kingdom Prospective Diabetes Study demonstrated that controlling the blood glucose, as measured by HbA1c and periodic blood glucose levels, reduced the development and progression of DR complications (190, 191), *post-hoc* analysis of the DCCT data has revealed that only 11% of the risk in retinopathy development could be attributed to HbA1c, with later studies showing significant, perhaps even greater impact of the glucose fluctuations (192), as well as the usage of insulin in type 2 DM with insulin resistance (193). As discussed above, the systemic microvascular disease that causes the progressive profound inflammatory injury is now recognized to exist in the prediabetic as well as the overt diabetic, especially among those demonstrating insulin resistance with the hyper and hypoglycemic variability (194). This accounts for a number of the drugs initially approved for the control of hyperglycemia, now having been recognized to have significant, but variable effects on the systemic as well as individual end-organ effects. This section will focus on those effects demonstrated within the retina and CNS.

### GLP-1 receptor agonists

Glucagon-like peptide-1 (GLP-1) receptor agonists (e.g. Liraglutide, Semaglutide, Dulaglutide, Extendin-4) have been demonstrated to pass the blood-brain barrier wherein they affect the neural tissue through the basic anti-apoptotic, antioxidant, and neurotrophic effects of GLP-1 receptor activation (195). This results in activation of both the protein kinase A (PKA) and phosphatylinositol-3-kinase (PI-3K) pathways, the results of which influence insulin activation in adipose tissue and also result in the transcription of genes responsible for antioxidant, anti-apoptotic, neurotropic, and anti-inflammatory effects (195–197). GLP-1 receptor activation results in reduction of the accumulation of intracellular reactive oxygen species (ROS) within microglia with increased production of NO (improving vascular autoregulation) as well as increased levels of antioxidant glutathione peroxidase and superoxide dismutase-1 (196). Furthermore GLP-1, *via* its effect of decreasing caspase 3 & 7 activity, also inhibits microglial production of TNFa, IL-1B, and IL-6, resulting in reduced insulin resistance systemically and neurally (198, 199). While glucose utilization, in general within neurons, occurs primarily *via* insulin-independent glucose transporter-3 (GLUT-3), there is known to be considerable variation within the brain. The forebrain, cerebral cortex, and hippocampus show co-expression of the insulin-dependent glucose transporter-4 and within these areas GLP-1 receptor activation appears to decrease the insulin resistance and perhaps even reverse it. In animal models GLP-1 receptor activation has been demonstrated to improve cognitive functions by increasing glucose utilization (200). In clinical studies, however the results have been mixed, although the studies have remained small with varying individual traits that may cloud the recognition of efficacy (196, 201). The observed effects upon ischemic injury demonstrated within the studies appear predominantly within the penumbra of infarcts, and therefore future studies should be undertaken as the examination methods and understanding improve. Moreover, the new glucagon-like peptide-1 receptor (GLP-1R) combinations, developed together with the gastric inhibitory polypeptide (GIP) and glucagon agonists, also need well-planned clinical trials (196, 202).

### Biguanides

The biguanides (e.g. Metformin) in animal studies have demonstrated glucose-reducing effects through activation of AMP activated protein kinase (AMPK), with reduction in clinical studies of proinflammatory cytokines due to inhibition of the activation of macrophage nuclear factor kappa B (κ) that results in reduction in IL-1B, IL-6, and TNFa (140). However, the clinical studies have been mixed in the demonstration of such inflammatory marker reductions (140). Some clinical studies have demonstrated reductions in the deposition of advanced glycosylated end products within vascular smooth muscle (203, 204), with the UKPDS demonstrating reduction in the risk of stroke. However, at best the evidence is conflicting, with similar conflicting results also of the alpha-glucosidase inhibitors in which modestly reduced CRP levels are associated with some improvements demonstrated in coronary flow, but no data with regard to neurovascular effects (140). Therefore, to understand better what effect the bioguanides may have upon the neuropathology requires further clinical testing (140).

### Thiazolidinediones

The thiazolidinedione class of antidiabetic drugs are known to activate the peroxisome proliferator-activated receptor γ (PPARγ). Following many types of acute and chronic neural injury, PPARγ serves as a master gatekeeper of cytoprotective responses to stress, improving the chances of cellular survival and recovery of homeostatic equilibrium (205). In the retinal ganglion cells PPARγ activation has been demonstrated, in animal studies, to result in neuroprotection against the glutamate-induced cytotoxicity (206). In the acute injury phase PPARγ inhibits the NFκB pathway to mitigate inflammation and stimulates the Nrf2/ARE axis to reduce oxidative stress. During the chronic phase of acute brain injuries, PPARγ activation culminates in the repair of gray and white matter, with preservation of the blood-brain barrier, reconstruction of the neurovascular unit, resolution of inflammation, and with apparent long-term functional recovery (205). Rosiglitazone has demonstrated reduced inflammatory markers and superoxide anion production with inhibition of ubiquitin-proteasome activity in atherosclerotic plaques of T2DM patients (140), while Pioglitazone systemically has demonstrated reduced adipose tissue macrophage activity resulting in decreased pro-inflammatory cytokines (IL-6, IL-1B) in neutrophils, macrophages and dendritic cells of peripheral organs (140).

### Sulfonylureas

Regarding the clinical neurologic effects of the sulfonylureas, information does not appear supportive of a beneficial effect. The UK General Practice Research Database revealed that, compared with metformin monotherapy, treatment with the sulfonylureas resulted in a significant 24% greater stroke occurrence (207, 208), while the Veterans Administration Data Base demonstrated an 11% increased incidence (209). Analysis of a multitude of additional, randomized controlled trials has also revealed an increased stroke incidence compared with metformin, DPP-4 inhibitors, GLP-1 receptor agonists, glitazones or with just insulin (210). The Glinide group of drugs exhibit mechanisms similar to that of the sulfonylureas; Mitiglinide has demonstrated some reduction in oxidative stress levels and inflammatory markers IL-6, IL-18, and TNF-a in T2DM, but studies of clinical neurologic effects are lacking (140).

### Dipeptidyl peptidase inhibitors

Dipeptidyl peptidase (DPP-4) inhibitors such as Sitagliptin have been demonstrated to reduce CRP, TNF-a, TLR-2, TLR-4, IKKB, and CCR-2 systemically with improvement in inflammation and endothelial function, independent of the hypoglycemic activity, in T2DM patients with associated coronary artery disease (140). The DPP-4 inhibitors, however, unlike the GLP-1 activators, cannot pass the BBB or BRB, but interestingly their effect within the central nervous system appears to be through associated increased endogenous GLP-1 levels (140). To date, no randomized prospective studies have been conducted on a sufficient number of humans to study the neurocognitive or retinal effects.

### Sodium glucose cotransporter 2 inhibitors

SGLT-2 inhibitors (SGLT-2i) or “Flozins” (Empagliflozin, Canagliflozin, Dapagliflozin, Ertugliflozin, Sotagliflozin)) represent a new class of systemic, oral treatment of hyperglycemia. Plasma glucose is lowered by inhibiting the reabsorption of glucose within the kidneys. Clinical studies have demonstrated that SGLT2i lead to a decrease in insulin secretion, enhanced beta-cell function and insulin sensitivity, with a demonstrated decrease of renin-angiotensin and sympathetic vasomotor system activity that has provided a demonstrated additive advantage for retinopathy (211).

The SGLT-2i are lipid-soluble with varying ability to penetrate the BRB and BBB. In addition they have variable affinity for the SGLT1 receptors as well, demonstrating protection against ischemia/reperfusion brain damage (211). SGLT2i show an anti-inflammatory and anti-atherosclerotic effect with inhibition of AchE, and as well, mitigate oxidative stress, exerting a protective effect on the neurovascular unit, blood-brain barrier, pericytes, astrocytes, microglia, and oligodendrocytes (211). Among the group, Sotagliflozin demonstrates the greatest affinity to SGLT1 receptors, and therefore is often called a “dual SGLT1/SGLT2 inhibitor”; however, it is the newest Flozin and has as yet not been used in diabetic patients on a large scale (211). Empaglifozin has the highest SGLT2 selectivity and greatest brain/serum ratio and has been demonstrated to significantly increase the level of cerebral BDNF, which modulates neurotransmission and ensures growth, survival, and plasticity of neurons (212). SGLT2i’s also in animal studies also demonstrate enhanced synaptogenesis and angiogenesis, con- tributing to neuroplasticity (213) a mechanism essential in neurorehabilitation.

The effects of SGLT1 *versus* SGLT2 inhibition in neuroprotection varies among the drugs evaluated and studies (214), as the sites of the receptors vary considerably within the brain parenchymal locations and demonstrate varied functional outcomes. Certainly, the efficacy requires further investigation and delineation, which is of increasing clinical relevance due to the availability of SGLT1/2 dual inhibitors, such as sotagliflozin, Furthermore there is now *in vivo* evidence indicating that SGLT2 may have an influence on the composition of the cerebrospinal fluid (CSF), whose role in the pathology of neurodegenerative disorders provides a new direction for research (215).

In conclusion, patients with insulin-resistant type2 diabetes as well as pre-diabetes are at increased risk and have inferior outcomes than non-insulin resistant or non-hyperglycemic subjects. Glycemic control achieved with the use of sulfonylureas or insulin was reported to increase the incidence of stroke in older observational studies but not in recent prospective, randomized, controlled trials. It is likely that an imbalance in the unmeasured clinical factors is responsible for the results in these observational studies. Insulin use itself may represent a more potent marker for insulin-resistance, and hence a better predictor of risk for stroke or progressive small vessel ischemic disease than HbA1c. The use of metformin, a-glucosidase inhibitors and DPP-4 inhibitors have thus far in studies demonstrated conflicting clinical evidence, as noted above while the utilization of pioglitazone and long acting GLP-1 receptor agonists have been associated with a decreased incidence of ischemic cerebral vascular disease. Once dementia due to small vessel disease has developed in diabetic patients, it appears not to be reversed by tighter glycemia control, but rather may be aggravated even more by the variability of the serum glucose levels and hypoinsulinemia as have been demonstrated within the retina (194). This has raised concern within some the few clinical studies of SGLT2i; although less likely to cause hypoglycaemia than insulin, they have been reported to be associated with increased hypoglycaemia and secondary neurologic AE’s when used in combination with metformin and sulfonylureas (216). Interestingly, intranasal insulin, has been proposed as an alternative adjunct therapy to improve cognitive function and memory (in doses that do not cause hypoglycemia), and certainly should be evaluated.

## Current and future treatments of diabetic retinal and brain neurovascular disease

The treatment of CNS WMHI’s of presumed vascular origin primarily has been limited to lifestyle modifications and risk factor management, and certainly, given the associations, it is imperative to target macro as well as microvascular health strategies throughout life. Consumption of tuna/nonfried fish and the components of the Mediterranean diet have been reported to be associated with less WMHIs (56, 214), while higher plasma omega-3 polyunsaturated fatty acids (abundant in both diets) are associated with loaa of WMHI-associated executive function. However more recent analysis of population-based clinical trials have revealed considerable problems with such statistical population analysis and have recommended additional, randomization methods to validate the associations.

There is growing evidence that, within the retina, the components of the renin angiotensin system (RAS) occur in neurons, glia, and blood vessels and that activation plays an important role in the pathogenesis of diabetic retinopathy (217). RAS blockage, using either an angiotensin converting enzyme (ACE) inhibitor or one of the angiotensin II type 1 (AT1) receptor blockers (e.g. candesartan which demonstrates the best diffusion across the blood-brain barrier), has produced a neuroprotective effect in an animal model following brain focal ischemia, while valsartan was able to prolong the survival of astrocytes and reduce glial activation in a retinal hypoxia model (63, 215–219). In clinical trials utilizing ACE inhibitors there has been some controversy whether in type II diabetics, with or without hypertension, there is retinal protection over and above the blood pressure lowering effect (220); however *post hoc* analysis of the trials did reveal a significant benefit after adjusting the data for the duration of diabetes, HbA1c, and the systolic blood pressure. In type 1 diabetics with mild or moderate DR, candesartan, however, was not effective in preventing DR progression (220), while in another clinical study a reduction in the WMHI number and volume was demonstrated (221).

Aspirin has been the most commonly utilized anti-platelet agent recommended in diabetics for both prevention of cardiovascular events (including stroke); however, no contemporary trial, conducted specifically in patients with diabetes, has thus far provided definitive data showing benefit for primary stroke prevention or end-organ microvascular progressive injury (222). In two studies meta-analysis of trials that included diabetic patients, only a modest, 14% decline was demonstrated in ischemic stroke but with a 34% significant increase in hemorrhagic stroke (223, 224). While steroids certainly cannot be administered systemically among the diabetic population at large, the recognition that systemic inflammatory effects are centrally involved should certainly promote the consideration of other treatments such as the tyrosine-protein kinase receptor (TIE-2) path activators or angiopoietin-2 blockers or the kallikrein-kinin inhibitors. Hopefully such trials are on the horizon.

In the future, however, clinical research must be directed toward, not just the prevention of further progression of the neurovascular occlusion and blood-brain or blood-retinal barrier destruction, but also the repair. Evaluation of therapeutics for both diabetics and prediabetics that focus on the neurovascular unit are desperately needed because of the recognized onset and progression of dementia, as well as vision loss in this exploding population. However, this has been limited by the current imaging evaluation and functional testing. As outlined by Bicker (225) the search for innovative, effective, and safe treatments for CNS disorders is challenging and impeded by multiple factors, including the multifactorial mechanisms underlying these disorders, as well as the limitations of currently available BBB imaging methods. *In vitro* BBB models offer a partial depiction of *in vivo* reality but can be costly and labor intensive and are insufficient for drug screening, as they lack reproducibility and/or standardization (dynamic models and microfluidic platforms). The retina certainly offers clinical imaging and functional testing that surpass that of radiologic brain imaging and cognitive function analysis. We suggest that approaches to the treatment of the neurovascular degenerative processes as well as repair of the microvascular injury within the neurovascular unit, should, at least initially utilize retina derived outcomes. Most likely such strategies will require a combination of therapies to achieve the desired multiplicity of outcomes with improved diagnostic methods to evaluate. This section of the review will summarize currently recognized treatments and outcomes achieved with recommendations for future directions where possible.

### Neurovascular unit responses of inflammation to the treatment of oxidative stress

Increased oxidative stress is known to play a central role in the pathogenesis of many diseases such as atherosclerosis, vascular inflammation, and endothelial dysfunction and is thought to play a role in the progression of prediabetes to diabetes (140) as well as in the onset and progression of the neurovascular complications.

Nicotinamide adenine dinucleotide phosphate (NADPH) oxidases appear to play a central role in redox-signaling cascades, under normal physiologic conditions producing reactive oxygen species (ROS) primarily for functions of innate immunity and the production of necessary hormones (226). Because of their overarching role in ROS generation in the diabetes state, NADPH oxidases appear as an attractive target for future therapeutic strategies. Animal studies have demonstrated Resveratrol’s ability to suppress neuroinflammation by inhibiting NADPH oxidase and attenuating NF-κB-induced expression of iNOS (inducible nitric oxide synthase), COX-2 (cyclo-oxygenase-2, an inducible inflammatory enzyme), and sPLA2 (secretory phospholipase A2) (227–229). Resveratrol also has demonstrated some restoration of the microRNA functions discussed above, thus making it appear as an attractive treatment, although human studies of diabetics are currently lacking. The polyamine oxidase pathways, including a key enzyme, spermidine oxidase, also have been demonstrated in animal and human studies to be associated with lipid peroxidation and retinal neurovascular injury from oxidative stress with the production of toxic byproducts, such as acrolein, a highly reactive unsaturated aldehyde that is also found as a contaminant in food, air, and water. Vitreous samples in human studies (of FDP-lysine, a biomarker of acrolein, formed when acrolein conjugates with lysine residues) have demonstrated correlations with retinopathy but only with the more severe forms (230). Scavengers of such toxins appear to include a number of nitrogen (amino)-containing, current oral medications including hydralazine, carnosine, aminoguanidine, pyridoxamine, edaravone, and phenelzine. A potent acrolein scavenger, 2-hydrazino-4,6-dimethylpyrimidine (2-HDP), was observed to significantly decrease the presence of FDP-lysine in the retina of diabetic rats with reduced activation of microglia and inflammation markers and with attenuation of Müller cell gliosis, improved oxidative stress markers, and improved visual function (231). Certainly clinical studies focused on the impact of acrolein-scavenging agents are needed. While defense against oxidative stress is thought to be a major event involved in the inhibition of the harmful effects upon Müller cells and neurons, compounds with both antioxidant activity and such amino toxin-scavenging capacity would appear to have a greater impact (230).

Coenzyme Q10 (CoQ10) is an endogenous antioxidant within the inner membrane of the mitochondria of the endothelia and smooth muscle of the microvasculature. Recent animal studies have provided evidence for the potential pleiotropic and anti-inflammatory role of CoQ10 by demonstrating its ability to inhibit induced interleukin-6 (IL-6), tumor necrosis factor-α (TNF-α), and nuclear factor-κB (NF-κB) expression (232, 233).

Reduced levels of CoQ10 in the aging and those with DM have been reported (234). CoQ10 supplementation influences the cholinergic system and in clinical studies appears to protect cholinergic neurons in patients with Alzheimer’s disease, as well as improving cardiovascular function (235). In addition, CoQ10 appears to reduce hyperglycemia (236–238), thereby protecting against endothelial abnormalities and dysfunction in DM (237) while reducing oxidative stress (237, 239). In a study of middle-aged, diabetic rats, evaluated cognitive function and learning memory were improved. However, in clinical studies of CoZ10 have suggested supplementation may improve learning and memory deficits induced by diabetes (240). However in reviewing patient evaluations, variations in the results were suggested to be because of penetration variability within various brain regions and pathways resulting in different types of learning, memory, and cognitive processing which would cause significant variation in the CNS function (240). Therefore, the CNS functional evaluation of CoQ10 supplementation remains to be adequately evaluated by clinical studies as extensively discussed above for the many systemic therapies.

The hyperglycemia induced increase in intracellular reactive oxygen species that leads to the secondary microvascular endothelia and pericyte alterations with leukocyte adhesion resulting in microvascular occlusion and endothelial injury, insulin resistance, protein/macromolecule glycation, and inhibition of NO synthesis. The NO inhibition, in turn, alters the microvascular reactivity required for the normal autoregulatory control of blood flow. In human diabetic retinas after administration of oral pentoxifylline an interesting phenomenon was observed of improvement in the capillary WBC velocity (148), although the mechanism, whether through improved WBC deformability or from reduced endothelial production of ICAM’s and VCAM’s, is unknown as well as the effects on the BRB and microvascular occlusion. Pentoxifylline has demonstrated a beneficial effect overall of reduced systemic inflammatory markers (241), but the clinical effects within the retina and brain remain to be investigated.

### Neurovascular protection associated with anti-inflammatory treatment

In the diabetic or prediabetic patient, as discussed above, there is pervasive, chronic systemic low-grade inflammation which is reflected by elevated levels of serum cytokines such as TNF-α, CRP, IL-6, iL-18, IL-1B receptor antagonist, and C5a (140, 154, 242) along with elevated serum WBC’s (granulocytes, monocytes) and elevated ICAM-1 and VCAM-1 but with a reduction in the protective effects of brain-derived neurotrophic factor (BDNF) (155). While this systemic inflammatory state is noted to be associated with the development and progression of retinopathy (243), of cerebral WMHI’s (244) and small vessel disease heart failure (140), the mechanisms remain to be defined as, indeed, the etiology is complex (245). The dysfunction of the neurovascular unit in diabetes includes several intracellular signaling cascades resulting in proinflammatory cascades that can either be helpful, neutral or detrimental to cell survival. Since there is now a recognized mutual relationship between systemic inflammation and several metabolic reactions that occur within the retina and CNS, interest is developing to improve the effects of immunomodulatory agents upon the disease process of the microvasculature (140, 246). For example, IL-1β has been demonstrated to induce pericyte apoptosis *via* NF-κB activation with increased endothelial permeability (84, 171) and occlusive degeneration of retinal capillaries in DR (84, 247). Therefore, limiting the IL-1β-triggered inflammatory processes, perhaps by blocking its receptor could offer a valid therapeutic approach.

A new method of potentially limiting the end results of systemic inflammation is through modification of the semaphorin molecular signaling cascades. The semaphorins are extracellular signaling molecules with recent animal study evidence indicating the role in regulating adipogenesis, adipose inflammation, and diabetic complications including microvascular permeability in multiple organs (248). Within the five semaphorin classes in vertebrates, the Class-3 family (Sema3A-3G) appear the most pertinent. Sema3E has been identified in a dietary mouse model as a regulator of adipose tissue macrophage accumulation in obesity with inflammation that contributes to systemic insulin resistance (249). Systemic antibody inhibition to Sema3E markedly reduced the tissue inflammation and improved insulin resistance, suggesting that a Sema3E peptide vaccine may provide a therapeutic potential for obesity as well as modifying diabetic complications in general.

In addition, several semaphorins have been noted to be involved directly in the retinopathy complications. Sema3A is induced in ischemic retinal ganglion cells in response to IL-1 after vascular injury in animal models (250) and, promoting endothelial apoptosis (251), appears to prevent revascularization, early in the course when the neurons are still salvageable (100). In the same model Sema3A also appears to promote apoptosis in retinal neurons (252), contributing to the retinal progressive neurodegeneration. A Sema3A-neutralizing antibody alleviated both the increased permeability and neurodegeneration while also facilitating normal revascularization of the inner retina (253) suggesting a potential therapeutic course for early staged retinopathy. However, as yet no clinical studies are begun. In the same model Sema3E, also produced by ischemic neurons, appeared to normalize revascularization and suppresses extra retinal vascular outgrowth (254). Sema3E expression is reduced in the vitreous of DM patients with more advanced retinopathy, suggesting suppression (254). Stimulation of Sema3E may offer an additional, adjunctive therapy if such protein mediators are able to sufficiently pass the BRB and BBB.

Toll-like receptors (TLRs) are a class of pattern recognition receptors on macrophages and microglia within neural tissue that normally recognize a diverse set of pathogen-associated molecules not present in the normal host. When activated, TLRs signal downstream pathways that are also known to activate the NF-κB and IL-1β generation paths, which, with the amplification by astrocytes, result in the generation of pro-inflammatory cytokines and chemokines. TLRs have been found that are also capable of responding to endogenously derived molecules, such as components released from necrotic cells or molecules that may be formed as a consequence of other pathologic mechanisms. More specifically TLR-4 polymorphisms have been identified associated with Type 2 DM raising the question as to whether these receptors additionally contribute to the inflammatory processes associated with the resultant neurodegeneration (245, 255). Targeting these molecules, therefore, could reveal a number of molecular and pharmacologic potentials to modulate the resultant inflammation. As an example, minocycline, due to its ability to readily penetrate the CNS and demonstrate anti-inflammatory properties, has appeared well-suited for such retinal and CNS disorders (27, 256). Minocycline blocks microglial activation in response to a variety of inflammatory stimuli by inhibiting TLR-2 and TLR4 signaling and several MAP kinases. In a rat model of diabetic retinopathy, minocycline treatment repressed the release of pro-inflammatory cytokines IL-1β, and TNF-α with a concomitant reduction in caspase-3 mediated apoptosis (257). In patients with diabetic macula edema oral minocycline has been demonstrated to reduce abnormal vascular permeability with a modest improvement in visual acuity and OCT defined tissue edema (258). More recently another antibiotic, azithromycin (also with immunomodulatory capabilities) has been noted to block RGC death in a retinal ischemia/reperfusion mouse model by modifying the inflammatory state (259). Taken together, these findings underscore the importance of further testing to define if such toll receptor blocking agents may be utilized as microglia-directed immunotherapy in human disease.

Advanced glycation end products (AGE’s) are proteins or lipids that are formed through non-enzymatic glycation as a result of being exposed to hyperglycemic conditions and are intensively related to the microvascular and macrovascular complications (22, 140). AGEs bind to and activate the AGE receptor (RAGE) on the surface of microglia, astrocytes, vascular endothelial cells, and neurons, initiating a resultant proinflammatory response. This acts *via* heterodimerization with TLR-4 to stimulate microglia in the production of pro-IL-1B, pro-IL-18, and NLRP-3 (260). In this context, the measurement of soluble RAGE has been postulated as a valuable biomarker of these molecular events (261). Blocking the interaction with RAGE has been demonstrated in animal models to reduce the production of the resultant proinflammatory mediators (262), suggesting potential for human therapeutic intervention. However, while the circulating peripheral proinflammatory markers have been associated with CNS WMHIs (136, 244) there is considerable variability associated with retinopathy (156), requiring further investigation.

Extensive research has been conducted studying the events resulting in neuron death that occur with an acute stroke and how the pathways may be used as potential therapeutic targets for treating the occurrence of small as well as large vessel chronic ischemia. Activation of the mitochondrial permeability transition pore (MPTP) has been associated with an increased permeability associated with stroke that results in neuronal death. Cyclosporine A, a widely used immunosuppressant, has been recently shown to possess neuroprotective properties through its ability to block the MPTP. This has recently stimulated research on its use to reduce the severity of cell death with stroke or small vessel ischemic conditions (263).

Histone acetylation is increased within the retina when exposed to elevated glucose due to a decreased activity of histone deacetylase (HDAC) that has been demonstrated to result in an increased expression of pro-inflammatory cytokines from Müller cells (264). The favorable effects of both Valproate and Memantine on the BRB and BBB are attributed to the suppression of NF-κB activity by hyperacetylation of HDAC (220). This facilitates its transcription and release from astrocytes. Memantine appears to exert additional anti-inflammatory effects by also regulating the microglial activation (265).

The activation of the microglial PPARγ., as noted above, ameliorates the disruption of the neural injury after an ischemic insult through activation of the NFκB pathway restoring the local levels of claudin-5 (266) that also assist in restoration of the BBB due to an apparent anti-inflammatory action induced of macrophages and microglia (205, 242, 267). Various compounds with different therapeutic mechanisms appear to offer protection against neuroinflammation and progressive neurodegeneration through PPARγ activation (e.g. the anti-dyslipidemic, and the hypoglycemic thiazolidinedione agents), that, certainly require further investigation (220, 268). There also have been demonstrated anti-angiogenic effects within the retina. While trials of Fenofibrate (a PPARα agonist) have not shown beneficial effects upon lipid exudates present within the retina, a significant reduction in progression of diabetic retinopathy has been demonstrated (269, 270). Synthesized compounds also have recently been developed that act as dual PPARa & PPARγ agonists with apparent improved glucose and lipid metabolism. While Muraglitazar was discontinued during phase 3 trials due to adverse vascular events, Tesaglitazar and Aleglitazar appear promising along with new pan-PPAR agonists (271).

The Muller cell release of a multiplicity of inflammatory enzymes in response to the hyperglycemic and ischemic stimuli of the endoplasmic reticulum has been demonstrated due to the activation and release of transcription factor 4 (ATF4). This is blocked in animal studies by the local or systemic administration of the small molecule, 4-phenylbutyrate (PBA) (272). It is notable that PBA has been used many years for the treatment of patients with sickle-cell disease, thalassemia, and cystic fibrosis (272), and, as it is a small molecule that penetrates the blood-retinal and blood-brain barrier, its potential effects upon diabetes neuronal complications appears to warrant future investigation (272).

Targeting activation of microglia (175, 273–275) and mast cells (175, 276, 277) has gained increasing traction as a potential therapeutic avenue for the treatment of neuralvascular degeneration due to inflammation induced small vessel disease. The interleukins are the prototypic inflammatory cytokines, which often operate in concert with other inflammatory enzymes such as TNF-α. Antagonists to Il-1 have been shown to reduce levels of inflammation as well as improve the glycemic control in T2DM patients *via* increased B-cell recovered insulin secretion (278). Since the retinal and CNS neuroinflammatory lesion progression in the past has been associated with the adipose inflammatory resistance to insulin, perhaps IL-6 or IL-1 receptor antagonists may provide interesting future treatments. Tocilizumab, a humanized monoclonal antibody against the IL-6 receptor, blocking activation, has been shown to improve insulin sensitivity in rheumatoid arthritis (RA) patients (279). With regard to TNF-α antagonists, many phase IV studies have demonstrated that TNF-α blockers (antibodies) consistently decrease levels of the inflammatory cytokine, CRP, and have demonstrated protective effects against cardiovascular events in RA patients (140). TNF-α is certainly recognized as a primary modulator of the pathology encountered in a number of neurologic disorders and has gathered significant attention in the recent past, although the serious side effects of commercial TNF-α inhibitors have resulted in concerns regarding their efficacy, and treatments such as monoclonal antibody solutions most likely will not be considered for administration to diabetics en-mass. However, there is increasing effort to extract the TNF-α inhibitor/blocker effects within a host of natural products, phytochemicals, and nutraceuticals that may prove to represent alternative treatments. Many natural phytochemicals have been demonstrated to inhibit inflammation with concomitant amelioration of oxidative stress and, together with lesser side effects, have been suggested they be offered as alternative treatments for a number of inherited neurodegenerations (e.g. Alzheimer’s and Parkinson’s) (280), and as such, may also warrant consideration for neurodegenerative complications of DM, although the effect upon the microvasculature is unknown.

Growth differentiation factor-15 (GDF-15) belongs to the transforming growth factor-β family and is also known as “macrophage inhibitory cytokine-1”. Its expression is enhanced in response to tissue ischemia (186). Investigations have demonstrated a positive association between GDF-15 serum concentrations and diabetes risk of retinopathy with higher levels associated in proportion with the retinopathy severity grade level (187). However, the mechanisms in the pathogenesis are not clear, although possible explanations have suggested that GDF-15 inhibits the oxidative stress and endothelial dysfunction (188, 281) with involvement in the inflammatory processes as well (187). Further research is anticipated (140).

During the inflammatory process, as discussed above, the endothelial excitation is a primary factor that results, not only in increased vascular permeability and eventually endothelial apoptosis, but also the secretion of intercellular adhesion molecules that adhere to leukocyte integrins, resulting in the complex VLA-4, the primary surface reactant on the leukocyte that adheres the cell to the endothelial derived ICAM-1 and VCAM-1. As discussed above the adhesion of the leukocyte results in microvascular occlusion with breakdown of the leukocyte resulting in endothelial leakage (147). The humanized anti-VLA-4 mAb (natalizumab) has demonstrated clinical activity in a number of autoimmune diseases (282), and its investigation in diabetes, especially for early retinopathy, appears warranted, although the administration of monoclonal antibodies remains a severe barrier for mass treatment. Similarly, the activation of the protein kinase C (PKC) pathway appears integrated in the genesis as well as progression of diabetic retinopathy. The PKC-B inhibitor, Ruboxistourin is the most researched PKC inhibitor in cellular, animal and human studies, demonstrating normalization of retinal blood flow in diabetic patients (283). The PKC DRS studies determined the drug to significantly delay the occurrence of visual impairment but could not reduce the progression of the more severe NPDR stages to proliferative retinopathy or alter the severity of significant macular edema (52, 284). Similar to other singular pathway therapies, further trials are required to examine potential combinations that will more adequately address the complexities of the interactions.

Targeting microRNA dysregulation, which results in a number of the aspects of the diabetic microvascular injury (but in particular endothelial dysfunction) certainly renders this as a target for monitoring and warrants further investigation for possible intervention (183, 285). Human prospective studies are necessary to evaluate the effects within the microvasculature, perhaps initiating with the retina, with evaluation of the microvascular density, flow, leakage with then the evaluation of the resultant neural cellular structural abnormalities and function pursued as they become more easily defined. With regard to the regulation of this signaling in the brain, insulin and insulin-like growth factor-1 (IGF-1), activating PI3K-Akt signaling that protects the BBB properties of the endothelial cells through the phosphorylation of GSK-3β, has been demonstrated to increase the expression of multiple microRNAs that influence the function and integrity of the endothelial barrier (286). Upregulation of serum IGF-1 concentration has been associated with acceleration of DR (287); however, the Randomized Clinical Trials (RCTS) resulted in conflicting results of the inhibition by Octreotide on progression of DR, although this was predominantly oriented toward the more advanced retinopathy forms (288). Further investigation certainly appears warranted for intervention during the earlier DR stages, when the processes are reversible as discussed above.

### Combination therapeutic approaches

Because of the multiplicity of pathways involved in the development and progression of the neurovascular injury, as discussed above, Chalke and Kale have suggested that a combination approach appears worthy to consider and have identified a number of potential medications (289). Citicoline, an intermediate in the synthesis of phosphatidylcholine, has been used to treat CNS degenerations such as Alzheimer’s and Parkinson’s with demonstrated promotion of repair and growth of cell membranes with neurotransmitter improvements. The neuroprotective action appears *via* interference with the deposition of B-amyloid resulting in improved functional outcomes (290). In addition, it has been evaluated in animal studies as a neuroprotective agent in the treatment of glaucoma (291, 292) wherein it appears to reduce neuronal degeneration by reducing caspace 3 production, and to reduce reactive gliosis by decreasing GFAP levels with some anti-inflammatory effects through the prevention of the upregulation of NF-kB. Chalke and Kale, in their review (289) suggest combining Citicoline with Resveratrol demonstrating the ability as discussed above to suppress Caspase 3 and caspase 8, with neuroinflammation inhibition of NADPH oxidase and the attenuation of NF-κB-induced expression of iNOS, COX-2, and sPLA2 (152, 229, 293) as well as restoring of mitochondrial SIRT-1. Combination drug studies have demonstrated stroke penumbra reduction in animals (289), but clinical studies have yet to begin.

Chalke and Kale have also suggested combining Duloxetine with N-Acetyl Cysteine. Duloxetine is a serotonin and norepinephrine inhibitor that demonstrates anti-oxidative and anti-inflammatory properties, as well as the ability to modulate the expression of angiogenic and neurotrophic factors that assist in attenuating the DR neuronal and vascular effects in animal studies (294). N-acetyl cysteine, as discussed above, is an antioxidant, scavenging reactive oxidative species with restoration of superoxide dismutase and inhibition of NF-kB, reducing inflammation and pericyte loss. Therefore, the combination, through adjunctive pathways may be beneficial to early DR in preventing progression, but as discussed, further clinical studies are necessary.

Chalke and Kane also considered the combination of CD5-2 with an angiopoietin-2 inhibitor. CD5-2 is an oligonucleotide that increases vascular endothelial cadherin, restoring pericyte integrity of the endothelial cell barrier, as well as attenuating VEGF signaling in the resultant inflammation. The angiopoietin-2 inhibitor interacts with the TIE-2 tyrosine kinase receptors improving endothelial health and adhesion. The combination is suggested as a potential improved alternative treatment for the progressive breakdown of the BRB, wherein Anti-VEGF agents combined with steroids, although reducing edema, have demonstrated little functional testing improvement (289).

## Conclusion

Systemic inflammatory mechanisms certainly appear active in a number of the recognized neurodegenerative processes associated with DM, with microglia and astrocytes as the primary contributors to the inflammation. Normally quiescent, such activated cellular components begin to secrete cytotoxic substances that contribute to neuronal death (195, 231). Therapeutic approaches, therefore, would logically aim to modulate the sensor/transducer/effector functions of the innate immune system to reduce TLRs, NF-κB, TNF-α, IL-6, and IL-1B respectively, or, as discussed above in combination with the administration of protective effectors such as PEDF (pigment epithelial-derived factor), BDNF (brain-derived neurotrophic factor), CNTF (ciliary neurotrophic factor), and NGF (nerve growth factor). However, these approaches also face numerous challenges with respect to timing, efficacy, and safety. Animal models certainly suggest that targeting the involved inflammatory pathways may be effective in preventing disease progression but perhaps are limited in healing the prior neuroparenchymal injury. Pursuing this certainly would require identification and treatment of at-risk patients prior to the development of overt signs or severe symptoms, and therefore clinical biomarkers of subclinical pathology would thus be of great utility for identifying such at risk patients and for monitoring the efficacy of treatments. To be clinically effective, systemic anti-inflammatory therapies will have to gain access to the CNS and retinal parenchyma through the BBB and must be tuned toward target specific cells and pathways that are quantitatively important in the disease pathogenesis of humans, certainly looking at neurons as endpoints as well as other cellular contributions and vascular components. In addition, as discussed above, due to the complex nature of these processes and the numerous cell types involved, it may require a combination of drugs to confer a therapeutic benefit, which, therefore most probably will require new clinical trial designs.

This was recently expressed as well in an editorial reviewing neuroprotection, neuroenhancement and neuroregneration within the retina and optic nerve (295). Despite the availability of treatments to mitigate some of the most prevalent disease- driving processes, including intraocular pressure reduction in patients with glaucoma or anti-VEGF therapy in patients with neovascular age-related macular degeneration, many patients experience continued neuronal death and vision loss even during seemingly adequate treatment. Further, there is a lack of interventions proven to alter the clinical trajectory of less prevalent conditions. Indeed, this review of preclinical literature from the past 20 years highlights a dizzying array of molecules, pathways, and therapeutic approaches that protect RGCs and photoreceptors in animal models. Why have none of these emerged as viable treatments for patients? The factors that have limited clinical translation include the use of animal models that incompletely incorporate key features of human diseases (including species lacking a macula or collagenous lamina cribrosa), species differences in molecular and cellular signals that drive neurodegeneration, differential pharmacodynamics and kinetics between animal models and humans, redundancy within signaling pathways, and challenges in identifying druggable targets. Study designs for neuroprotection treatment trials of slowly progressing diseases certainly require new design strategies. For instance, temporal clustering of structural and functional testing modalities and the use of trend-based, rather than event-based, outcomes may have greater sensitivity for detecting neuroprotective treatment effects, requiring reduced sample sizes and shorter observation periods (296–298). Furthermore, continuously advancing diagnostic technologies, including swept-source OCT, OCT angiography, and the potential to directly visualize apoptotic neurons, afford increasingly precise measures of relatively small degrees of neurodegenerative worsening. Therefore, substantial optimism accompanies the numerous neuroprotection treatment trials currently in progress.

New methods of imaging of the focal neuronal apoptosis and inflammation are in progress (299, 300) and hopefully will enable earlier detection of neuronal injury when the apoptosis process is reversible and empower initiation of treatments before death and severe atrophy. New methods of imaging with non-invasive laser scanned retina OCT (OCTangio), also appear to enable identification of focal microvascular occlusive events that have been noted to occur prior to the traditional lesions of retinopathy (98) along with analysis of retinal capillary white blood cell flows utilizing the non-invasive blue field, entoptic phenomenon (57). Whether these preclude or are associated with the focal neuronal apoptosis is for evaluation in the future as new methods also now allow the functional testing to be overlaid onto the neuronal and microvascular occlusion lesion imaging within the retina. It is imperative, therefore, that diabetics and prediabetics be screened regularly utilizing both functional testing of the central field with fixation control and under conditions of reduced contrast and luminance to define earlier occurrences and with retinal imaging utilizing one or another of the methods described above. Given the current recognition in the diabetic and prediabetic of early neuronal, pericyte, and endothelial apoptosis occurring due to the imbalance of the pro-apoptotic and protective enzymatic processes (301), it is imperative as well that the brain be imaged regularly with newer MRI, as well as PET scanning, which probe microstructural integrity, molecular biology, as well as activation patterns that do appear to provide new insights into brain-behavior relationships (302) for all but the subcortical microinfarcts (112). In this regard, the measurement of vascular resistance using a pulsatility index by transcranial doppler ultrasonography (reflecting vascular resistance of small vessels) has been shown to be increased in association overall with the presence of white matter lesions and the degree of coalescence (303). Whether this also reflects increased large artery stiffness from AGE plaque, however, is unknown, and furthermore, the technique appears not to demonstrate sufficient focality to associate with individual lesion size and distribution. An important question still remains as to whether neuroimaging itself can distinguish and quantify mixed pathogenic features with such accuracy that it could produce markers of onset and evolution of the cerebral ischemic lesions and the response to therapeutic interventions (112). Neuroimaging techniques that appear most readily applicable to the study of small vessel pathology include time-of-flight and ultra-high-field susceptibility-weighted imaging that discerns structural characteristics of the small vessels. The progressive neurodegeneration, caused or accelerated by the progressive oxidative and inflammatory processes discussed above, appears complex and demands means of monitoring, either by serum measures of CRP, IL-1, IL-6, TNF-α, MCP-1, BDNF, or others mentioned, or possibly by the application of non-invasive Ramon intraocular hyperspectral imaging to depict quantitative characteristics of the cytokines within the retina or in the overlying vitreous.

Once discovered and tracked, however, the difficulty still remains as how to address the progressive neurovascular complex degenerative processes. This review certainly has demonstrated that, although each factor alone may be a significant contributor to the pathologic progression, the interactions among these factors are likely more complex than expected. Locally within the eye, there is early, but significant evidence that the non-invasive, periodic application of micropulsed therapeutic laser (without photocoagulation injury) is associated with significant improvement of structural and functional measurements (105). However, now that the systemic nature of these autoimmune, inflammatory neurovascular processes is recognized with ultimate multi-organ injury, this must be addressed as well with systemic therapeutic means that penetrate the BBB and BRB, and with diagnostic means of assessing the therapeutic outcomes.

Thus, there are many novel questions that researchers must answer. Basic science models of clinical neural small vessel disease have certainly sought to improve our understanding of causal factors, pathophysiologic natural history, and potential intervention and treatment targets. However, to date, animal models have not described the full spectrum of phenomena observed in human small vessel disease and neuronal injury of the retina and brain which certainly demands more complex association studies (109) including genetic variability that has also been shown to be influential (304). In all regards, the treatment of the neuro-vascular degeneration that occurs and progresses among the exploding population with pre-diabetes and diabetes, even with the optimum glycemic control, remains unsatisfactory and therefore demands an urgent need of finding treatment methods or combinations thereof to retard and potentially halt and reverse the progression of this functionally devastating, systemic disorder. Certainly, such treatments must be entertained as early as possible in the course with guidance of interventions to achieve these goals. Imaging and functional testing of such lesions in the retina now surpass those in the brain for the detection and grading of the neurovascular evaluations within the early disease process when reversible with restorative healing but also must be evaluated among the more severe stages to prevent the severe blinding and cognitive crippling outcomes that currently manifest in so many sufferers. In all cases, the treatments must be associated with rehabilitation methods that understand the integrated visual field and the visual problems associated with task failure in order to develop the assistive work-arounds together with adequate instruction and training to achieve adequate patient engagement and task management.

## Author contributions

All authors listed have made a substantial, direct, and intellectual contribution to the work and approved it for publication.

## Conflict of interest

Author SSi is the CEO of Sinclair Technologies, LLC and a consultant in Molecular Targeting Technologies, LLC. Author SSc is a member of the Medical Advisory Board in Salix Pharmaceuticals and Arkay Therapeutics and is employed at Speaker’s Bureau for Salix Pharmaceuticals, Janssen Pharmaceuticals, Boehringer Ingelheim, Eli Lilly, and Merck Pharmaceuticals.

The remaining authors declare that the research was conducted in the absence of any commercial or financial relationships that could be construed as a potential conflict of interest.

## Publisher’s note

All claims expressed in this article are solely those of the authors and do not necessarily represent those of their affiliated organizations, or those of the publisher, the editors and the reviewers. Any product that may be evaluated in this article, or claim that may be made by its manufacturer, is not guaranteed or endorsed by the publisher.
